# Removal of organic micropollutants from water by adsorption on thermo-plasma expanded graphite encapsulated into calcium alginate

**DOI:** 10.1007/s40201-023-00876-9

**Published:** 2023-08-23

**Authors:** Marco Cuccarese, Stijn W. H. Van Hulle, Ignazio M. Mancini, Salvatore Masi, Donatella Caniani

**Affiliations:** 1 Scuola di Ingegneria, Università degli Studi della Basilicata, viale dell’Ateneo Lucano n.10, 85100 Potenza, Italy; 2 Department of Green Chemistry and Technology, Faculty of Bioscience Engineering, Universiteit Gent, Gr.Karel.de Goedelaan 5, 8500 Kortrijk, Belgium

**Keywords:** Adsorption, Fixed-bed, Micropollutants, Thermo-plasma expanded graphite, Water treatment

## Abstract

Nowadays, public concern is focused on the degradation of water quality. For this reason, the development of innovative technologies for water treatment in view of (micro)pollutant removal is important. Indeed, organic (micro)pollutants, such as pharmaceuticals, herbicides, pesticides and plasticizers at concentration levels of μg L^−1^ or even ng L^−1^ are hardly removed during conventional wastewater treatment. In view of this, thermo-plasma expanded graphite, a light-weight innovative material in the form of a powder, was encapsulated into calcium alginate to obtain a granular form useful as filtration and adsorption material for removal of different pollutants. The produced material was used to remove atrazine, bisphenol-A, 17-α-ethinylestradiol and carbamazepine (at concentration levels of 125, 250 and 500 µg L^−1^) by top-down filtration. The effect of flow rate, bed depth and adsorbent composition was evaluated based on breakthrough curves. The experimental data was analysed with the Adams-Bohart model in view of scale-up. Under optimal conditions, removal and adsorption capacity of respectively about 21%, 21%, 38%,42%, 43 µg g^−1^, 44 µg g^−1^, 37 µg g^−1^ and 87 µg g^−1^ were obtained for atrazine, bisphenol, 17-α ethinylestradiol and carbamazepine when using 0.12 g of thermo-plasma expanded graphite to treat 200 mL at 500 µg L^−1^ (for each compound) of solution obtaining at contact time of 20 min. The granular form of TPEG obtained (GTPEG) by entrapping in calcium alginate results to have a good adsorbent property for the removal of carbamazepine, atrazine, bisphenol A and 17-α ethinylestradiol from water at concentration levels between 250 and 500 μg L^−1^. Promising results confirm the adsorbent properties of TPEG and push-up us to investigate on its application and improve of its performance by evaluating different entrapping materials.

**Supplementary Information:**

The online version contains supplementary material available at 10.1007/s40201-023-00876-9.

## Introduction

Water is a precious resource and freshwater on the Earth is very rare and only 0.2% of the total water is directly accessible for human consumption [36]. Its distribution in the world is inequal and some countries face freshwater scarcity. The future increase of world population expected in the next years will even increase the problem associated to the freshwater scarcity [49]. In this context, the source of freshwater needs protection and intensive treatment and reuse of wastewater should be aimed for [15]. Wastewater cannot be directly discharged into surface water because can affect its quality. Nevertheless, globally 80% of the produced wastewater is discharged in surface water without treatment [47]. For this reason, an increase of wastewater treatment plans (WWTP) is required to preserve the quality of the water, although this alone will not be enough as a typical WWTP is not able to remove emerging pollutants such as herbicides, plasticizers, pesticides and pharmaceutical products that are still detected in WWTP effluent [13, 14, 26, 45, 46].

Indeed, innovative treatment needs to be introduced to remove these pollutants. To achieve this goal, operational aspects of the conventional activate sludge process were looked upon, such as increasing the hydraulic retention time [16] or sludge retention time [40] even if it involves an increase of operation cost. Alternative biological treatment technologies, such as a membrane bioreactor [11] and a biofilter [17, 23], were investigated and good results were obtained. Also technologies for tertiary treatment such as advanced oxidation process, adsorption and membrane filtration were tested [31, 37, 39, 48]. With respect to adsorption, commercial available activated carbon is the most common used adsorbent [12, 39], although several innovative adsorbents, such as biochar [10, 30, 42], activated carbon from waste [7], carbon nanotubes [24], natural polymers [3, 21, 34] and graphitic or graphenic substances [2, 8, 51] are being investigated or used.

In this work, thermo-plasma expanded graphite (TPEG) produced by an innovative process was used as adsorbent material to remove different types of organic micropollutants from water by adsorption. That material could represent a substitute of activated carbon as adsorbent material with higher adsorption capacity because the thermo-plasma expansion guarantees a significant exfoliation associated to a significant increase of the activated area of the material. The process confers low apparent density and the material floats on the water due to its light-weight characteristics and as such TPEG was entrapped into calcium alginate polymers by in-situ cross-linking. Therefore, the entrapping step results to be necessary to obtain a form of TPEG usable as filter medium for water treatment and remediation. The method used was inspired by physical entrapment of enzyme for biosensor’s production [28, 41] and already used in water treatment [18, 29, 43]. Recently, the method is used to prepare adsorptive material that can be used in filtration systems [22, 25, 33]. The entrapment process was optimized to produce a granular TPEG (GTPEG) heavier than water and the material obtained was characterized by SEM, FT-IR and BET analysis. The adsorption process was then characterized to evaluate the removal of carbamazepine, atrazine, 17-α ethinylestradiol and bisphenol A. Carbamazepine and 17-α ethinylestradiol are pharmaceuticals, while bisphenol-A is a plasticizers and atrazine a herbicide. All four micropollutants have hazardous effect on human life. The effect of the flow rate, bed depth, initial concentration, GTPEG composition on the removal treatment was investigated as well as the long-term stability of the entrapped TPEG. The pollutants selected as target compound represent emerging pollutants of water. Therefore, the developing of processes and materials able to remove that kind of micropollutants pollutants from water represents a significant challenge for the science. The major novelties of the work are represented by different points. The first one is the developing of a process of preparation of the granular form of the thermo-plasma expanded graphite (GTPEG) and the investigation of the use of GTPEG as adsorbent material for the removal of the cited emerging micropollutants from water by filtration. The entrapping of TPEG into granular polymer resulted to be an easy way able to guarantee the use of that material as filter medium for treatment of water by filtration on adsorbent fixed-bed. The second novelty is that the material developed here (GTPEG) could represent a substitute of activated carbon for treatment into WWTPs to overcome the challenge of the removal of the micropollutants. The third novelty is represented by the fact that the entrapment process developed and used here could be transferred to other fine powder adsorbent material that do not precipitate into the water and cannot be easily used as filter medium.

## Materials and methods

### Materials

Sodium alginate and calcium chloride were purchased from Carlo Erba reagents All chemicals were of analytical grade (purity > 98%) and used without further purification. TPEG was purchased from Innograf s.r.l. Atrazine, bisphenol-A, carbamazepine and 17-α ethinylestradiol standards were purchased from Sigma Aldrich. All solutions were prepared in deionised water (electrical conductivity below 5 µS cm^−1^). A saturated solution of the individuals micropollutants was prepared by adding an amount that equals three times the solubility of the compound to 1 L of deionised water. This saturated solution was vigorously stirred for three hours. The solution was then filtered on Rotilabo type 601 cellulose filter (Carl Roth, 5–13 µm of retention range) to remove undissolved particles. Then, individual solutions were diluted with water to obtain a stock solution of 1 mg L^−1^ and stored at 4 °C to avoid degradation. Every two weeks working solutions were renewed. Before each experiment, working solutions were mixed and diluted to obtain the required concentration.

### Preparation of granular thermo-plasma expanded graphite

To prepare granular thermo-plasma expanded graphite, 20 g of sodium alginate was added to 1 L of distilled water and stirred until a homogeneous gelatinous solution was obtained. Then, TPEG was added to the solution and stirred for 24 h to obtain a homogeneous solution. Different amounts of TPEG (2.5%, 5%, 7.5% and 10%, amount expressed as function of total weight of sodium alginate) were added to the solution to test the effect of the composition of adsorbent material on the filtration operation and estimate the optimal amount. When the homogeneous solution was obtained, it was gradually transferred into a 250 mL separating funnel and dripped into 1 L of solution of CaCl_2_ (2%) which was gently stirred. The presence of Ca^2+^ ions results in cross-linking of the alginate chains and formation of insoluble spheres where TPEG is entrapped. The spheres where then recovered by filtration and dried in the oven at 105 °C for 24 h. The granular thermo-plasma expanded graphite (GTPEG) obtained was used as adsorbent material for further testing. The GTPEG prepared by adding 7.5% and 10% of TPEG were not useful for filtration because of its low density which resulted in floatation during the water treatment tests. As such, 5% of TPEG was estimated as the maximal amount that can be added to 1 L of water to obtain a suitable water treatment material. GTPEG obtained by different relative amount of TPEG were denominated GTPEG 2.5%, GTPEG 5%, GTPEG 7.5% and GTPEG 10% respectively.

### Characterization of granular thermo-plasma expanded graphite

The GTPEG obtained were characterized by SEM, BET and FT-IR analysis. SEM images was obtained by using a high-resolution field emission scanning electronic microscopy (HR-FESEM), Auriga Zeiss model, at CNIS laboratory of University of Sapienza (Rome, Italy). FT-IR spectrum was obtained in the range 400–4000 cm^−1^ (16 cm^−1^ of resolution) by using a ThermoNicolet 5700 FT-IR spectrophotometer (Thermo Fischer Scientific, https://www.thermofisher.com/be/en/home.html). For the FT-IR analysis, GTPEG 2.5%, 5%, 7.5% and 10% samples were analyzed to compare these samples with each other and with individual alginate and TPEG spectrum. All the sample was measured in the form of KBr pellet, prepared by mixing 0.2 g of sample to 20 g of KBr (stored in the oven at 105 °C to eliminate trace of humidity), crushedby hand in a mortar and pressed at 9 tons cm^−2^. The characterization of material was done consistent with literature information [4, 6, 22, 52].

### Labscale experiments

#### Fixed bed column adsorption test for system optimisation

**Fig. 1:**
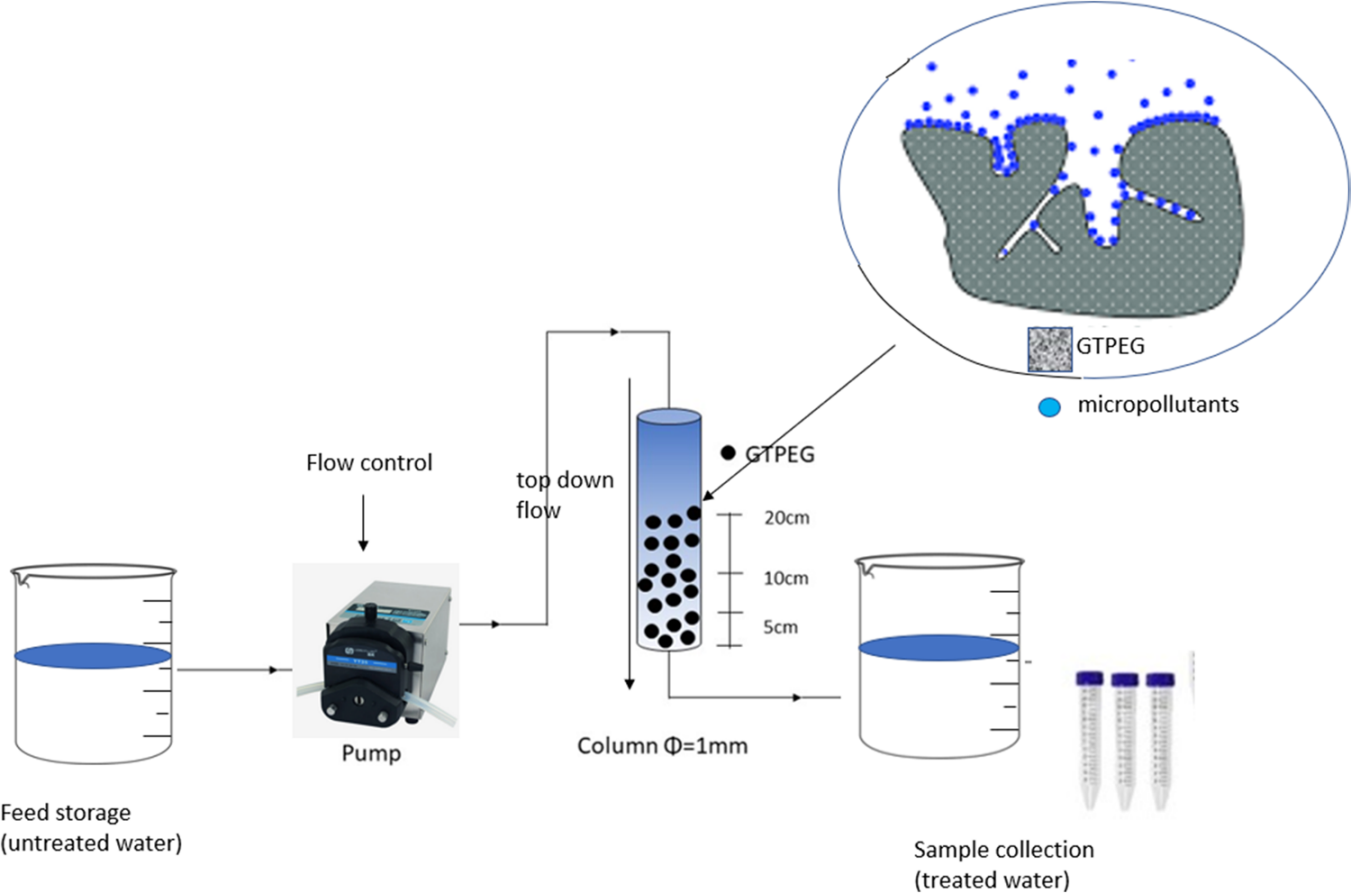
Schematic representation of lab-scale filtration plant

A 50 cm long glass burette of 1 cm of diameter was used as the column for all adsorption tests. A cotton filter was added to the bottom of the column as a support to avoid loss of adsorbent material. Prior of each experiment, the column was filled with wetted GTPEG and deionized water was passed through the column to avoid the formation of air bubbles. The -micropollutants solution was pumped through the column by a peristaltic pump connected by silicones tubes to the column and the flow was controlled by the valve at the bottom of the burette. For all the experiments a top-down flow was imposed and 200 mL of effluent collected every 10 mL for analysis. In the Fig. 1 is reported the schematization of experimental scheme.

The effect of the flow rate on the removal and/or breakthrough was evaluated by using 500 µg L-1 solutions of atrazine, bisphenol, 17-α ethinylestradiol and carbamazepine which were introduced at flow rates of 0.2, 1 and 2.7 mL min^−1^ to a 5 cm GTPEG 5% (2.4 g, therefore 0.12 g of TPEG) column. These flow rates correspond to contact times of 25 min, 5 min and 1.8 min respectively.

The effect of the bed depth on the removal and/or breakthrough was evaluated by using 500 µg L-1 solutions of atrazine, bisphenol, 17-α ethinylestradiol and carbamazepine which were introduced at flow rate of 1 mL min^−1^ to columns of 5 cm (2.4 g of GTPEG5% = 0.12 g of TPEG), 10 cm (4.8 g of GTPEG5% = 0.24 g of TPEG) and 20 cm (9.6 g of GTPEG5% = 0.48 g of TPEG). These conditions ensured a contact time of 5, 10 and 20 min respectively. For these test GTPEG 5% was used.

The effect of the initial concentration of micropollutants on the removal and/or breakthrough was evaluated in order to have information on the minimal concentration that can be treated by GTPEG. The evaluation was performed by using different initial concentrations of atrazine, bisphenol, 17-α ethinylestradiol and carbamazepine (125, 250 and 500 µg L-1) at a flow rate of of 1 mL min^−1^. A column height of 10 cm (4.8 g of GTPEG5% = 0.24 g of TPEG) was used to ensure a contact time of 10 min and linear velocity of 1 cm min^−1^. For all these test GTPEG 5%was used.

In order to have information about the influence of the composition of GTPEG, some tests were performed at a concentration level of 500 µg L^−1^ with a column height of 10 cm (4.8 g of GTPEG5% = 0.24 g of TPEG, 4.8 g of GTPEG2,5% = 0.12 g of TPEG) at a flow rate of 1 mL min^−1^ to ensure a contact time of 10 min. Granular alginate without TPEG was compared with GTPEG2.5% and GTPEG5%.

#### Leaching test

To verify that TPEG did not leach from the prepared granular material and the adsorption performance is not affected, a leaching test was performed with the material. The column was filled with 10 cm of GTPEG 5% and 5 L of deionized water was pumped through it at a flow rate of 100 mL min^−1^ and linear velocity of 10 cm min^−1^, an higher velocity of the tested condition that can carry on particles of TPEG if it were not entrapped very well into the alginate and on its surface. After water pumping, 200 mL of micropollutants solution at initial concentration of 500 µg L^−1^ was treated by pumping it through the above reported column at the flow rate of 1 mL min^−1^ (contact time 10 min and linear velocity of 1 cm min^−1^. Breakthrough curve, removal and adsorption capacity were evaluated and compared with that observed were no leaching test was applied.

### Data analysis of fixed bed column adsorption data

#### Removal efficiency and adsorption capacity

For all the test considered, breakthrough was reached after filtration of maximal 200 mL of micropollutant solution. To evaluate the time of the filtration, the Eq. 1 can be used:

1 $$T=\frac{V}{Q}$$

where T is the time of filtration (min), V is the volume of effluent (mL) and Q is the flow rate (mL min^−1^). The removal efficiency (R) for each compound was calculated based on the breakthrough curve, plotting the ratio of the concentration in the effluent (C_eff_) and influent (C_inf_) versus the treated volume (V). The removal efficiency was calculated as follows (considering that the maximal treated volume (V_max_) in this study is 200 ml):

2 $$R= \frac{{\int }_{0}^{Vmax}(1- {~}^{{C}_{eff}}\!\left/ \!{~}_{{C}_{inf}}\right.)dV}{{\int }_{0}^{Vmax}dV}$$

The amount of each pollutants that is adsorbed (W) was calculated by Eq. 3:

3 $$W=R \times ({C}_{influent}\times Vmax)$$

The adsorption capacity (q)was calculated by Eq. 4:

4 $$q=\frac{W}{m}$$

where m was the mass of the adsorbent material. In this work, the adsorption capacity was calculated by considering both the amount of TPEG into the GTPEG (because this is the actual adsorbent material) and the total amount of GTPEG used. All the data obtained were processed by using Microsoft Excel software.

#### Adams-Bohart model and Thomas model fitting

The experimental data obtained were further analysed with the Adams-Bohart model and Thomas model to have a fundamental understanding of the adsorption process in view of scale-up of the process [19, 20]. The Adams-Bohart model assumes that the adsorption rate is proportional to the residual capacity and the concentration of adsorbed micropollutants. Normally, this model can be applied well in the first stage of the adsorption when C_eff_/C_inf_ < 0.15. The Adams-Bohart model used for the description of the initial part of the breakthrough curve is expressed by Eq. 5:

5 $$\frac{{C}_{eff}}{{C}_{inf}}= {e}^{( K{C}_{inf}t-K{N}_{0}\frac{Z}{F})}$$

where K is the kinetic constant (L µg^−1^ min^−1^), t is the time (min), N_0_ is the saturation concentration (mass of adsorbate adsorbed for unit of volume of bed, µg L^−1^), Z is the bed depth of the column (cm) and F is the linear velocity (cm min^−1^). By plotting the natural logarithm of C_eff_/C_inf_ versus the time is possible to obtain the value of the kinetic constant and saturation concentration when bed depth and column section area are already known. After the determination of K and N_0_, evaluation of reactor dimension when done. Equation 5 can be transformed in Eq. 6:

6 $$ln\frac{{C}_{eff}}{{C}_{inf}}=K{C}_{inf}t-K{N}_{0}\frac{Z}{F}$$

If the breakthrough point is reached, the value of dependent variable of the Eq. 6 is zero, therefore Eq. 6 can be arranged in Eq. 7:

7 $$K{N}_{0}\frac{Z}{F}=K{C}_{inf}t$$

Equation can be rearranged in Eq. 8:

8 $$\frac{Z}{tF}=\frac{{C}_{inf}}{{N}_{0}}$$

By programming an excel sheet, it is possible evaluate one of variable Z, t or F by fixing all the other parameters. In this work, F was evaluated by fixing Z (10 m of GTPEG5%) for C_inf_ of 500 and 250 µg L^−1^ and assuming to reach the breakthrough in one day by treating 10,000 L of contaminated water (flow rate 10,000 L for day). After evaluation of F,se surface area of the reactor was calculated by the Eq. (9):

9 $$surface\;area\;reactor\;\left(s\right)=\frac{flow\;rate}F$$

By assuming to use a circular reactor, diameter of it was calculated by using the equation to calculate surface of circle. Dimension of reactor was calculated for all the micropollutants considered at initial concentration of 500 and 250 µg L^−1^. From the volume of the reactor, the mass of GTPEG necessary to fill the reactor was also calculated by considering the density of GTPEG (480 g dm^−3^).

The Thomas model is one of the most general and used methods in column performance theory. The model assumes Langmuir kinetics of adsorption–desorption and no axial dispersion is derived with the adsorption that the rate driving force obeys second-order reversible reaction kinetics. By using this model, it is possible to evaluate the adsorption capacity of the system. The linear form of the model is regulated by the Eq. 10.

10 $$\ln\left(\frac{C_{inf}}{C_{eff}}-1\right)=k_{Th}\cdot q_e\cdot\frac xv-k_{Th}\cdot C_{inf}\cdot t$$

where k_Th_ is the Thomas constant rate (µg^−1^ L min^−1^), q_e_ is the adsorption capacity of the system (µg g^−1^), x is the amount of the adsorbent material (g) and v is the flow rate (L min^−1^). From the Thomas model, after the calculation of kinetics constant and theoretical adsorption capacity, the necessary amount of adsorbent material was estimated by considering to treat contaminated water with initial concentration of 500 and 250 µg L^−1^ at the flow rate of 10,000 L for day. From the amount of GTPEG necessary for the treatment considered, volume and diameter of reactor was calculated by considering the density of GTPEG and a bed depth of 10 m.

### Analytical procedure

In order to quantify the concentration in the effluent of considered micropollutants (carbamazepine, bisphenol A, atrazine and 17-α ethinylestradiol) micro-liquid extraction was performed to transfer analytes from water to organic solvent, then GC–MS analysis was conducted. Therefore, 1 mL of dichloromethane was added to 20 mL of water sample and vigorously handly-shaken for 10 min. After the extraction, 500 µL of the organic phase was taken and transferred into a GC–MS vial. An aliquot of 1 µL of the sample was injected in the splitless mode by an Agilent 7683 Series autosampler. The temperature of injection was set on 250 °C and helium gas was used at mobile phase at flow rate of 13.9 mL min^−1^. The chromatographical separation was performed on a fused silica capillary (5% phenyl)-methyl polysiloxane HP-5MS column (30 m length, 0.25 mm I.D. and 0.25 µm film thickness). The initial column temperature was programmed at 100 °C and hold for 1 min, then raised to 270 °C with temperature rate of 10 °C min^−1^. The mass spectrometer was operated in negative electron-impact ionization (EI) mode at 70 eV. A solvent delay of 2.0 min was used to preserve the ion source. The MS transfer line temperature was set at 200 °C, while the MS source temperature was maintained at 230 °C. MS spectra were acquired in SIM mode using one target ion that were 200 for atrazine, 213 for bisphenol, 193 for carbamazepine and 296 for 17-α ethinylestradiol. The total run time of the analysis was about 10 min.

## Results and discussion

### Characterization of GTPEG

**Fig. 2:**
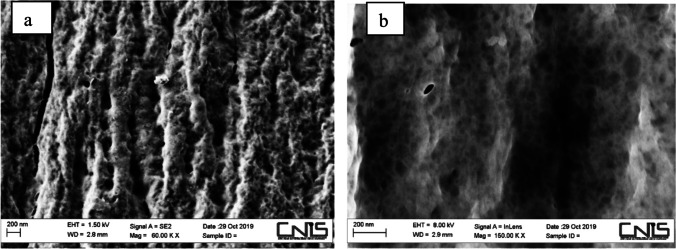
SEM images of GTPEG 5% at different magnification a) 60KX and b) 150 KX

In the Fig. 2, SEM images obtained for GTPEG 5% are reported. By the SEM analysis is possible to observe the fibrous and rough structure of GTPEG and the presence of pores useful for the adsorption process. Shape and pore size on the GTPEG surface seem to be heterogeneous and not uniform and it is a good indicator of presence of surface porosity, useful to react with adsorbate, as reported in literature [35].

**Fig. 3:**
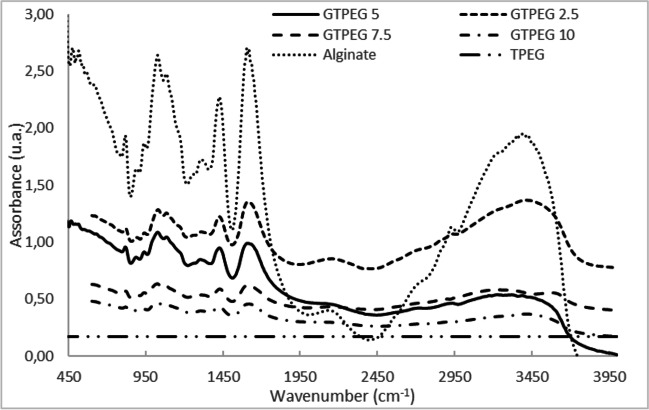
FT-IR spectrum acquired in the range 400–4000 cm^−1^ and resolution of 16 cm.^−1^

In the Fig. 3, the FT-IR spectrum obtained for granular alginate, TPEG, GTPEG 2.5%, 5%, 7.5% and 10% is reported. In the FT-IR spectrum the typical peaks associated with the carboxylic group of alginate at about 1000 cm^−1^ (associated to the C-O stretching vibration), 1400 cm^−1^ (associated to symmetric COO vibration) and 1600 cm^−1^ (associated to asymmetric COO vibration) [5, 38]. By increasing the amount of TPEG in GTPEG a decrease of the intensity of this peak is observed, proving that a higher relative amount of TPEG is entrapped into the alginate (note that into the spectrum of TPEG no peaks can be observed because it does not have functional group).

### Effect of flow rate

**Fig. 4:**
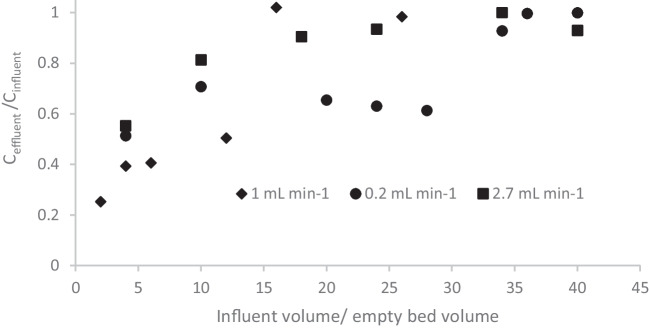
Breakthrough curves of carbamazepine at the flow rate of 0.2 mL min^−1^ (●), 1 mL min^−1^ (♦) and 2.7 mL min.^−1^ (■)

In the Fig. 4, the breakthrough curves of the carbamazepine for the three different flow rates (0.2, 1 and 2.7 mL min^−1^) are demonstrated. By decreasing the flow rate the exponential increase of the ratio C_eff_/C_inf_ was observed at higher breakthrough volume. For 0.2 mL min^−1^ it was observed at about 180 mL, about 90 mL for 1 mL min^−1^and 2.7 mL min^−1^.

In the Table 1 the typical parameter of adsorption on filtration bed are gathered for all the four compounds at the all experimented flow rate.. Breakthrough volume is considered as the volume when y axis reaches the value of about 1.

**Table 1:** Typical parameters of adsorption of considered micropollutants (initial concentration of 500 µg L^−1^) on 5 cm of filtration bed of GTPEG 5%

Flow rate (mL min^−1^)	q (µg g^−1^ GTPEG)	Removal (%)	Breakthrough volume (mL)
Atrazine			
0.2	186	22	180
1	127	15	110
2.7	106	13	100
Bisphenol A			
0.2	208	25	180
1	133	16	110
2.7	92	11	90
17-α ethinylestradiol			
0.2	292	35	180
1	128	15	110
2.7	111	13	80
Carbamazepine			
0.2	290	35	180
1	184	22	80
2.7	168	20	90

From Table 1 it becomes clear that by decreasing the flow rate an increase of adsorption capacity and removal is obtained as is also reported in literature [1, 19, 20, 22, 25, 27, 32, 33, 38, 50]. For example, for atrazine the adsorption capacity increased from 106 µg g^−1^ to 195 µg g^−1^ and the removal increase from 13 to 22%. The same effect was observed for the other micropollutants: for 17-α ethinylestradiol the removal efficiency even tripled (compared to a doubling for the other micropollutants).

By changing the flow rate also different breakthrough volumes were observed. Carbamazepine is the molecules with the highest affinity for GTPEG..

### Effect of bed depth

**Fig. 5:**
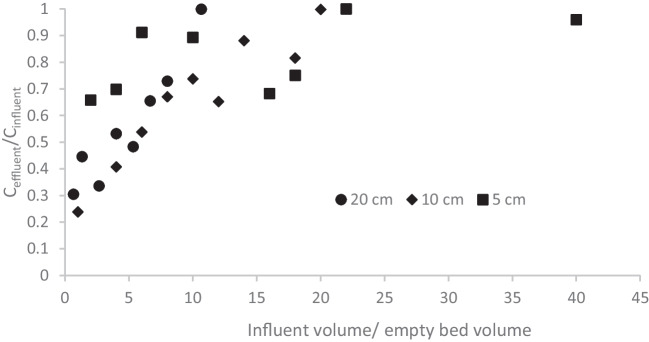
Breakthrough curves of 17-α ethinylestradiol (initial concentration of 500 µg L^−1^) at the flow rate of 1 mL min^−1^ filtered through a bed depth of 5 (■), 10 (♦) and 20 cm (●) of GTPEG 5%

In Fig. 5, the breakthrough curves of 17-α ethinylestradiol are presented as example of the effect of bed depth on the adsorption. By increasing the bed depth an increase of breakthrough volume was observed and the adsorption at the initial stage of the filtration increased. In the case of 17-α ethinylestradiol, a breakthrough volume of 200 mL was obtained for 20 cm and 10 cm bed height while a volume of 110 mL was obtained for 5 cm bed depth. By increasing the depth also a decrease of initial value of the ratio C_eff_/C_inf_ (increase of initial removal) was observed.

In the 2 the typical parameter of adsorption on filtration bed are gathered for all the four compounds at all the experimented bed depth tested to evidence the effect of this parameter.

**Fig. 6:**
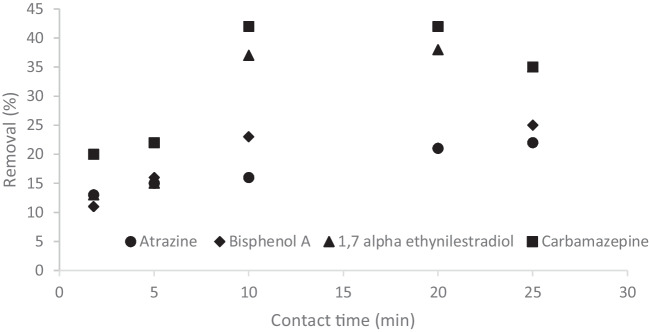
Effect of contact time on removal observed in the previous experiments

From Table 2 it can be seen that the increase of the bed height is the increase of the removal and breakthrough volume, as reported in literature [1, 19, 20, 22, 27, 33, 38] due to the higher amount of adsorbent material. The adsorption capacity increases when the increase of removal balances the increase of amount of adsorbent material. The bed depth of 20 cm ensures a contact time of 20 min that is the almost the same of the experiments conducted at 0.2 mL min ^−1^ on a column of 5 cm (25 min) and the results obtained confirms it. Therefore, a bed depth of 20 cm at 1 mL min^−1^ had the same performance of the filtration on 5 cm at 0.2 mL min^−1^ but the adsorption capacity is lower because more material is used and the denominator of adsorption capacity is higher. Because of the removal obtained at 1 mL min^−1^ with 20 cm of bed depth was the same of that one obtained at 0.2 mL min^−1^ and 5 cm of bed depth, bigger amount of water can be treated in the same time with same removal efficient. This solution can be used to face emergency situations and wasting of GTPEG is not the priority. by increasing the bed depth and flow rate. By the analysis of the effect of the bed depth on the adsoprtion, another interesting observation can be deduced. For bisphenol, 17-α ethinylestradiol and carbamazepine the same breakthrough volume and/or removal is observed by increasing the bed depth from 10 to 20 cm. This means that for contact times higher than 10 min, the contact time is not the limiting step of the adsorption process. Therefore the adsorption capacity does not increase and the removal is not affected. In the Fig. 6 the removal efficiency obtained at different contact time (includes the results obtained at different flow rate) are reported.

**Table 2:** Typical parameters of adsorption of considered micropollutants (initial concentration of 500 µg L^−1^) filtered through different bed depth of GTPEG 5% at 1 mL min^−1^

Bed depth (cm)	q (µg g^−1^ GTPEG)	Removal (%)	Breakthrough volume (mL)
Atrazine			
5	127	15	110
10	65	16	140
20	43	21	160
Bisphenol A			
5	133	16	110
10	96	23	140
20	44	21	160
17-α ethinylestradiol			
5	128	15	110
10	152	37	200
20	78	38	200
Carbamazepine			
5	184	22	80
10	175	42	200
20	87	42	200

The affinity of each compound for the GTPEG is as follows: atrazine > bisphenol > 17-α ethinylestradiol > carbamazepine.. Carbamazepine is the molecules with the highest affinity for GTPEG probably due to the higher number of aromatic rings compared to the other molecules. These aromatic rings can interact with the sp^2^ bonds of graphite. Atrazine has a lower affinity for GTPEG probably due to its lower number of aromatic rings and molecular weight, while 17-α ethinylestradiol could be more affine than bisphenol due its higher molecular weight, lower polarity and solubility.

### Effect of initial concentration

**Fig. 7:**
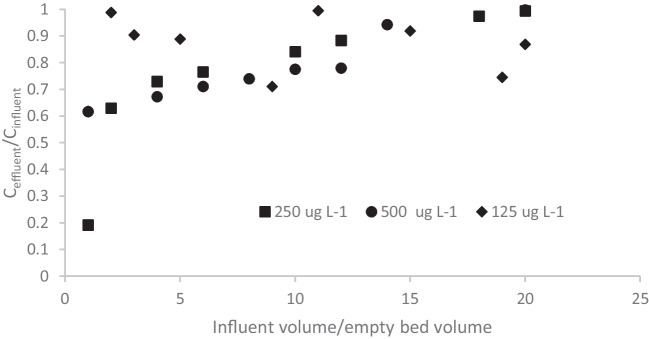
Breakthrough curves of bisphenol filtered on 10 cm of GTPEG 5% at the flow rate of 1 mL min^−1^. The initial concentration of the solution filtered were 125 (♦), 250 (■) and 500 µg L.^−1^ (●)

In Fig. 7, the breakthrough curves of bisphenol-A obtained at different initial concentrations (500, 250 and 125 µg L^−1^) using a column of 10 cm with GTPEG 5% at 1 mL min^−1^ are demosntrated. The decrease of initial concentration affects the breakthrough curves. At a concentration of 125 µg L^−1^ the influent and effluent concentrations are almost equal, even in the initial phase of the experiment. This means that little adsorption occurs at these low concentrations. adsorb at lower value of concentration. The other general considerations for the behavior observed at the value of 500 and 250 µg L^−1^ are reported in the next part of the test.

In the Table 3 the typical parameter of adsorption on filtration bed are gathered for all the four compounds at the all initial concentration tested to evidence the effect of this parameter.

**Table 3:** Typical parameters of adsorption of investigated micropollutants at different initial concentration filtered through 10 cm of GTPEG 5% at 1 mL min^−1^

Initial concentration (µg L^−1^)	q (µg g^−1^ GTPEG)	Removal (%)	Breakthrough volume (mL)
Atrazine			
125	28	7	10
250	74	18	190
500	65	16	140
Bisphenol A			
125	55	13	10
250	89	21	200
500	96	23	140
17-α ethinylestradiol			
125	56	14	10
250	139	33	200
500	153	37	200
Carbamazepine			
125	78	19	10
250	148	36	200
500	175	42	200

As normal observed in literature [9, 22, 27, 32, 33, 38], by decreasing the initial concentration a decrease of the adsorption capacity was observed for all the compounds considered. In the case of atrazine and bisphenol-A an elongation of breakthrough was observed due to the lower gradient of concentration at lower initial concentration. By decreasing the initial concentration to 250 µg L^−1^ from 500 µg L^−1^ no big variation in terms of removal was observed and it is a good indication to project a multi-filter system on series where an influent of initial concentration of about 500 µg L^−1^ for each compounds is filtered through the first filter and the resulting effluent is filtered through a second filter. In this way, by considering the removal observed in the previous experiments and reported in the previous table an influent of initial concentration of 500 µg L^−1^ could be transformed in an effluent of 347, 303, 211 and 187 µg L^−1^ for atrazine, bisphenol, 17-α ethinylestradiol and carbamazepine respectively. By adding another filter, an effluent of the concentration of 285, 238.5, 141 and 152 µg L^−1^ for atrazine, bisphenol, 17-α ethinylestradiol and carbamazepine respectively. A system of four filter could allow to reach a effluent of concentration of 238.5, 234, 122 and 124 µg L^−1^ for atrazine, bisphenol, 17-α ethinylestradiol and carbamazepine respectively.

From the analysis of breakthrough curves, lower value of the ratio C_eff_/C_inf_ (higher adsorption) were observed at the initial stage of the filtration because the adsorbent material has all its active sites free and the same amount of particles can be adsorbed for both the concentration of 500 and 250 µg L^−1^ and it means that in relative values the adsorption at 250 µg L^−1^ is higher at initial stage. After this initial stage, the value of the ratio C_eff_/C_inf_ is lower for the concentration of 500 µg L^−1^ because of higher gradient of concentration ensures higher adsorption. This behavior was observed for all the compounds tested.

### Effect of GTPEG composition

**Fig. 8:**
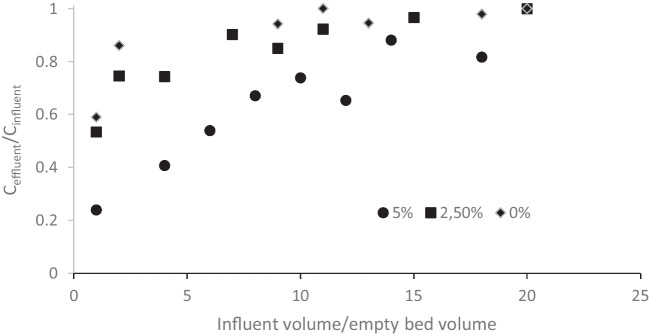
Breakthrough curves of 17-α ethinylestradiol (initial concentration 500 µg L^−1^) filtered on 10 cm of GTPEG 5% (●), GTPEG 2.5% (■) and GTPEG 0% (♦) at the flow rate of 1 mL min.^−1^

In the Fig. 8, the breakthrough curves of 17-α ethinylestradiol observed for different composition of GTPEG are shown. By increasing the concentration of TPEG into GTPEG an increase of adsorption was observed and as a consequence, higher breakthrough volumes were obtained. As previous reported, GTPEG 5% contains the higher amount of TPEG that can be added to obtain a material heavier than water (TPEG is light powder that float on water). Alginate contributes to the adsorption but, as next reported, its contribution to the adsorption capacity of GTPEG 5% was negligible.

In the Table 4 the adsorption results when using different concentrations of TPEG are summarized. In this case, also the value of the adsorption capacity calculated by considering only the adsorbent material (TPEG) is given.

**Table 4:** Typical parameters of adsorption for considered micropollutants for an adsorption column containing 10 cm of GTPEG with difference compositions operated with an influent concentration of 500 µg L^−1^ and an influent flow rate of 1 mL min^−1^

GTPEG content (%)	q* (µg g^−1^ GTPEG)	q (µg g^−1^ TPEG)	Removal (%)	Breakthrough volume (mL)
Atrazine				
0%	0.9	–	3	10
2.5%	1.1	25	3	10
5%	3	65	16	140
Bisphenol A				
0%	3	–	8	20
2.5%	4	92	11	70
5%	5	96	23	140
17-α ethinylestradiol				
0%	3	–	9	90
2.5%	5	133	16	150
5%	8	152	37	200
Carbamazepine				
0%	2	–	8	90
2.5%	7	175	21	200
5%	18	175	42	200

As logically expected, by decreasing the amount of TPEG used to produce GTPEG, a decrease of the removal and adsorption capacity were observed because lower amount of adsorbent material was present. The alginate can contribute to the adsorption because of its specific functional groups, detected in FT-IR conducted analysis, as reported in literature [33] but its contribute in terms of the adsorption capacity to the adsorption of GTPEG 5% is low. It was also negligible in the case of GTPEG 2.5% for the adsorption of 17-α ethinylestradiol and carbamazepine due to their higher affinity. The results obtained offer a new perspective for future studies: to find a different substrate to entrap TPEG heavier than calcium alginate to increase the amount of TPEG entrapped without affect the precipitation in the water.

### Leaching test

**Fig. 9:**
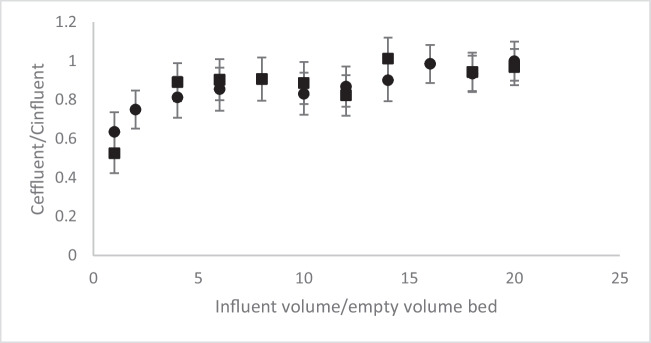
Breakthrough curves of atrazine (initial concentration 500 µg L^−1^) filtered on 10 cm of GTPEG 5% at the flow rate of 1 mL min^−1^ before (■) and after (●) leaching test

In the Fig. 9, the breakthrough curves of atrazine (initial concentration of 500 µg L^−1^) obtained with a bed height of 10 cm of GTPEG 5% at a flow rate of 1 mL min^−1^ before and after passing 5 l (500 times the bed volume) of water through the column (at a flow rate of 100 mL min^−1^). It is clear that no decrease of adsorption capacity or removal caused by leaching is observed and that as such it can be assumed that little leaching occurred. Therefore, it can be concluded that the method used to entrap the TPEG is a good choice although limited amount of it can be entrapped without affect precipitation in the water.

In the Table 5 the typical parameter of adsorption on filtration bed are demonstrated for all the four compounds at the all initial concentration tested to evidence the effect of this parameter.

**Table 5:** Typical parameters of adsorption of considered micropollutants at initial concentration of 500 µg L^−1^ filtered through 10 cm of GTPEG 5% at 1 mL min^−1^, before and after the leaking test. Standard deviation of adsorption capacity was approximately 10% for all the data reported in the table

Leaking test	q (µg g^−1^ GTPEG)	Removal (%)	Breakthrough volume (mL)
Atrazine			
Before	65	16	140
After	73	18	160
Bisphenol A			
Before	96	23	140
After	79	19	140
17-α ethinylestradiol			
Before	152	37	200
After	139	33	160
Carbamazepine			
Before	175	42	200
After	195	47	200

The results obtained from the leaking test demonstrate that no leak of TPEG happened and the adsorption capacity of the system is not affected, therefore the method used to entrap it results to be a good choice.

### Models fitting

The experimental data was further assessed with the Adams-Bohart model. With this model it is possible to have information about the kinetics of the process and the saturation concentration of the system. These parameters are useful to scale-up the system. In the Tables 6, 7 and 8 the correlation parameters of regression, kinetics constant and concentration of saturation obtained for all the compound at the different parameters tested are reported to compare the effect of their variations. Sometimes not excellent correlation was observed but it can be used as first analysis to have a confirm of general trend observed in experimental tests. For the atrazine and carbamazepine the data are reported in the Table S1 and S2 of online resources.

**Table 6:** Correlation parameter of regression, kinetics constant and saturation concentration (theorical and experimental) obtained by the Adams-Bohart fitting of experimental data obtained for bisphenol at the different conditions tested

Initial concentration (µg L^−1^)	Bed depth (cm)	Flow rate (mL min^−1^)	GTPEG%	Leaching test	K (L min^−1^ µg^−1^)	N_0_ (µg L^−1^)	N_0exp_ (µg L^−1^)	R^2^
Bisphenol A								
500	5	1	5	No	5.8·10^–6^	11,900	3200	0.93
500	5	0.2	5	No	2.6·10^–6^	3700	500	0.79
500	5	2.7	5	No	/	/	2200	0.04
500	10	1	5	No	5.8·10^–6^	9500	2300	0.93
500	20	1	5	No	5.6·10^–6^	9200	1000	0.81
500	10	1	2.5	No	/	/	300	0.01
500	10	1	5	Yes	1.8·10^–5^	8100	1900	0.89
250	10	1	5	No	1·10^–5^	4400	1100	0.47
125	10	1	5	No	/	/	300	0

**Table 7:** Correlation parameter of regression, kinetics constant and saturation concentration (theorical and experimental) obtained by the Adams-Bohart fitting of experimental data obtained for 17-α ethinylestradiol at the different conditions tested

Initial concentration (µg L^−1^)	Bed depth (cm)	Flow rate (mL min^−1^)	GTPEG%	Leaching test	K (L min^−1^ µg^−1^)	N_0_ (µg L^−1^)	N_0exp_ (µg L^−1^)	R^2^
17-α ethinylestradiol								
500	5	1	5	No	1.3·10^–5^	21,400	3100	0.80
500	5	0.2	5	No	2.4·10^–6^	7400	7000	0.31
500	5	2.7	5	No	5.6·10^–6^	21,500	2600	0.6
500	10	1	5	No	1.3·10^–5^	8700	3700	0.80
500	20	1	5	No	1.2·10^–5^	4600	1900	0.89
500	10	1	2.5	No	5.2·10^–5^	8100	1600	0.68
500	10	1	5	Yes	2.4·10^–5^	8600	3300	0.90
250	10	1	5	No	1.5·10^–5^	8400	1600	0.65
125	10	1	5	No	/	/	400	0

**Table 8:** Reactor diameter and volume and mass of GTPEG estimation for treatment of 10 m^3^ d^−1^ to remove carbamazepine, 17-α ethinylestradiol, bisphenol A and atrazine by adsorption on column of bed depth of 10 m of GTPEG5%

Initial concentration (µg L^−1^)	Diameter of the reactor (m)	Volume of the reactor (m^3^)	Mass of GTPEG (tons)
Atrazine			
250	5.7	255	130
500	8.3	540	268
Bisphenol A			
250	8.3	540	268
500	8.2	528	253
17-α ethinylestradiol			
250	6.1	292	142
500	8.5	567	275
Carbamazepine			
250	5.6	250	129
500	8.3	543	268

By the kinetic constants and saturation concentrations obtained from the Adams-Bohart model, general trends can be noticed. By increasing the flow rate, an increase of kinetic constant and a decrease of saturation concentration (except from calculated value of bisphenol and 17-α ethinylestradiol probably due to the model used) was observed for all the compound. This confirms that breakthrough is reached faster and lower amount of adsorbate saturates the system. By increasing the bed depth, a decrease of kinetics constant and adsorption capacity is observed, therefore the breakthrough is reached later as observed in experimental test. The decrease of saturation concentration can be explained by increase of amount of adsorbent used. The trend of saturation concentration is the same of the experimental observed. The decrease of initial concentration involves an increase of the kinetics of the process (except for carbamazepine). The saturation concentration decrease by decreasing the initial concentration of the influent (exception was observed for theoretical value of carbamazepine). As observed in the experimental section effect of initial concentration, by decreasing the concentration an elongation of breakthrough was observed but it does not involve a decrease of kinetics constant because of at the initial stage the removal was higher due to increase of relative numbers of active site respect pollutants molecules then the slope of the curve increases. The general trend observed in this work agrees with that already reported in literature [36, 40, 44]. By decreasing the amount of the TPEG entrapped, a decrease of the kinetics constant and saturation concentration was observed for 17-α ethinylestradiol but not for carbamazepine (from theoretical value). After the leaching test a small increase of the kinetic constant and small variations of the saturation concentration is noticed probably higher grade of hydration of the adsorbent material. As expected, the values of saturation concentration are not very close to experimental ones because of the model can be well adapted at the first stage of the breakthrough curves, but in this work we use it just for an estimations of the values of saturation concentration and kinetics that can be then compared with the values obtained from Thomas model, widely used for adsorption on fixed-bed. In the Table 10 the dimension of the reactor estimated by assuming to treat 10,000 L of contaminated water for day in a reactor of a bed depth of 10 m of GTPEG5%. Results evidence that a reactor of diameter of 8.3 and 8.5 m is necessary to treat water and remove carbamazepine, 17-α ethinylestradiol, bisphenol A and atrazine at initial concentration of 500 and 250 µg L^−1^ respectively at the flow rate of 10 m^3^ day^−1^ and bed depth of 10 m.

In the Tables 9 and 10 the correlation parameters of regression, kinetics constant and adsorption capacity obtained from the fit of experimental data with the Thomas model for atrazine and carbamazepine at the different parameters tested are reported to compare the effect of their variations. Furthermore, the experimental adsorption capacity is also reported to compare. In the Table S3 and S4 of online resources are reported the results obtained for bisphenol A and 17-α ethinylestradiol.

**Table 9:** Correlation parameter of regression, kinetics constant and adsorption capacity (theorical and experimental) obtained by the Thomas fitting of experimental data for atrazine at the different conditions tested

Initial concentration (µg L^−1^)	Bed depth (cm)	Flow rate (mL min^−1^)	GTPEG%	Leaching test	K (L min^−1^ µg^−1^)	q (µg g^−1^)	q_exp_ (µg g^−1^)	R^2^
Atrazine								
500	5	1	5	No	3.8·10^–5^	66	127	0.92
500	5	0.2	5	No	4.8·10^–6^	363	186	0.63
500	5	2.7	5	No	1.6·10^–4^	190	106	0.51
500	10	1	5	No	2.3·10^–5^	171	65	0.60
500	20	1	5	No	2.0·10^–5^	75	43	0.61
500	10	1	2.5	No	2.4·10^–5^	1146	25	0.34
500	10	1	5	Yes	4.2·10^–5^	23	73	0.80
250	10	1	5	No	7.7·10^–5^	13	74	0.67
125	10	1	5	No	/	/	28	0.2

**Table 10:** Correlation parameter of regression, kinetics constant and adsorption capacity (theorical and experimental) obtained by the Thomas fitting of experimental data for carbamazepine at the different conditions tested

Initial concentration (µg L^−1^)	Bed depth (cm)	Flow rate (mL min^−1^)	GTPEG%	Leaching test	K (L min^−1^ µg^−1^)	q (µg g^−1^)	q_exp_ (µg g^−1^)	R^2^
Carbamazepine								
500	5	1	5	No	9.5·10^–5^	163	184	0.98
500	5	0.2	5	No	1.5·10^–5^	236	290	0.57
500	5	2.7	5	No	6.6·10^–5^	127	168	0.68
500	10	1	5	No	9.9·10^–5^	136	175	0.77
500	20	1	5	No	7.8·10^–5^	62	87	0.74
500	10	1	2.5	No	5.9·10^–4^	48	175	0.61
500	10	1	5	Yes	8.7·10^–5^	160	195	0.90
250	10	1	5	No	1.1·10^–4^	51	148	0.62
125	10	1	5	No	1.3·10^–4^	44	78	0.32

By analyzing the data obtained by the Thomas model can be observed that the value of experimental and theorical adsorption capacity are close as expected because this model is widely used for all fixed-bed adsorption test. As observed by the Adams-Bohart model, by increasing the flow rate an increase of kinetic constant and decrease of adsorption capacity is observed. By increasing the bed depth, variation of kinetics constant is observed but the trend is different for each compound (decrease for atrazine and carbamazepine and increase for bisphenol A and 17-α ethinylestradiol). The adsorption capacity decrease by increasing the bed depth because the increase of removal does not balance the increase of amount of adsorbent material. By decreasing the initial concentration of the influent and amount of TPEG into adsorbent material a decrease of adsorption capacity and increase of kinetics constant is observed. Leaching test involves variation of kinetics constant and adsorption capacity due to the probably higher grade of hydration of adsorbent material, but as can be observed from experimental data, not loss of performance can be deduced. Also the general trend observed in this case agrees with that already reported in literature [36, 40]. In the Table 11 the dimension of the reactor estimated by assuming to treat 10,000 L of contaminated water for day in a reactor of a bed depth of 10 m of GTPEG5%. Results evidence that a reactor of diameter of 0.6 and 0.7 m is necessary to treat water and remove carbamazepine, 17-α ethinylestradiol, bisphenol A and atrazine at initial concentration of 500 and 250 µg L^−1^ respectively at the flow rate of 10 m^3^ day^−1^ and bed depth of 10 m. As expected, results are different from Adams-Bohart model because it is a good model for all fixed-bed system while Adams-Bohart model can be used just for the initial step of breakthrough curves and also because Thomas model can be used to estimate reactor dimension by assuming C_inf_/C_eff_ = 2. The better agreement between experimental and theoretical data predicted by Thomas model than Adams-Bohart suggests to consider the Thomas model as reference to scale-up of this system.

**Table 11:** Reactor volume and diameter and mass of GTPEG estimation for treatment of 10 m^3^ d^−1^ to remove carbamazepine, 17-α ethinylestradiol, bisphenol A and atrazine by adsorption on column of bed depth of 10 m of GTPEG5%

Initial concentration (µg L^−1^)	Mass of GTPEG (kg)	Volume of the reactor (m^3^)	Diameter of the reactor (m)
Atrazine			
500	584	1.2	0.4
250	3800	8.0	1
Bisphenol A			
500	1611	3.3	0.6
250	1800	3.8	0.7
17-α ethinylestradiol			
500	1611	3.3	0.6
250	908	1.9	0.5
Carbamazepine			
500	734	1.5	0.4
250	979	2.0	0.5

In the Table S5 reported in online resources, values obtained from Adams-Bohart, Thomas model and experimental of carbamazepine are reported for a fast comparison but as already mentioned, Thomas model is more indicated model for this system than Adams-Bohart. The value of saturation concentration was transformed into adsorption capacity by considering the density of GTPEG (480 g dm^3^).

### Literature comparison

In the Table S6 reported in online resources, relevant results obtained by adsorption of carbamazepine, 17-α ethinylestradiol, bisphenol A and atrazine on fixed bed are reported to have a faster comparison with results of this work. When comparison is done, it is important to remember the very low of amount of TPEG (adsorbent material) used to prepare the fixed bed in this work ( 0.24 g for 20 cm of bed depth represents the higher amount used) and the four pollutants are present in the same solution. Very few data in literature are available by considering a mix of these kind of pollutants in the same solution. Normally the conditions used in every work are different, but we can consider the results that we obtained comparable with that present in literature and it encourages us to continue to investigate on way to improve the use of this material.

## Conclusion

In this work a method to entrap an innovative adsorbent material (TPEG) was optimized and demonstrated. The granular form of TPEG obtained (GTPEG) results to have a good adsorbent property for the removal of carbamazepine, atrazine, bisphenol A and 17-α ethinylestradiol from water at concentration levels between 250 and 500 µg L^−1^. Good removal, about 40% for carbamazepine and 17-α ethinylestradiol and about 20% for atrazine and bisphenol A, was obtained by using a very low amount of TPEG (5% as weight relative to total alginate weight, GTPEG5%) and a low contact time (10 min). Furthermore, in the work it was demonstrated that experimental parameters such as flow rate, bed depth and composition of TPEG can be optimized to increase the removal and adsorption capacity. As example, the adsorption capacity of GTPEG can be increased from 111 to 292 µg g^−1^ for the 17-α ethinylestradiol, from 106 to 186 µg g^−1^ for the atrazine, from 92 to 208 µg g^−1^ for the bisphenol A, from 168 to 290 µg g^−1^ for the carbamazepine, by decreasing the flow rate from 2.7 to 0.2 mL min^−1^. Furthermore, the removal percentage can be increased from 15 to 38% for the 17-α ethinylestradiol, from 15 to 21% for the atrazine, from 16 to 21% for the bisphenol A, from 22 to 42% for the carbamazepine, by increasing the bed depth from 5 to 20 cm. Significant increases were observed also by increasing the TPEG percentage into the granular material prepared. Increase into the range of 10–20% were observed by doubling the content of TPEG. A systematic investigation was done to give information about the influence of the experimental parameters on the process and theoretical models (Thomas and Adams-Bohart) were used to confirms the influence observed and to estimate the dimension of the reactor for a scale-up of the process. By considering the models results 1611 kg of GTPEG into a reactor of diameter of 0.6 m (10 m of length) were necessary to treat 10 m^3^ d^−1^ of wastewater with initial concentration of 500 µg L^−1^ of each pollutant. These promising results confirm the adsorbent properties of TPEG and push-up us to investigate on its application and improve of its performance. To use an entrapping agent heavier than alginate can be useful to increase the amount of TPEG entrapped and to be sure to obtain a granular form of TPEG heavier than water and useful as fixed-bed adsorbent material could be the next step for the develop of the material as filter medium.

### Supplementary Information

Below is the link to the electronic supplementary material.

Supplementary file1 (DOCX 36 KB)

## Data Availability

The datasets used and/or analysed during the current study are available from the corresponding author on reasonable request.
