# Supplementary material for: Removal of organic micropollutants from water by adsorption on thermo-plasma expanded graphite encapsulated into calcium alginate
Source: J Environ Health Sci Eng. 2023 Aug 23;21(2):497–512. doi: 10.1007/s40201-023-00876-9 (PMC10584748; doi:10.1007/s40201-023-00876-9)
Supplement: Supplementary file 1 — Supplementary file1 (DOCX 36 KB) [file 40201_2023_876_MOESM1_ESM.docx]

**Removal of organic micropollutants from water by adsorption on thermo-plasma expanded graphite encapsulated into calcium alginate**

*Marco Cuccarese^*^, Stijn W.H. Van Hulle^**^, Ignazio M. Mancini^*^, Salvatore Masi^*^, Donatella Caniani^*^*

** Università degli Studi della Basilicata, Scuola di Ingegneria, viale dell’Ateneo Lucano n.10, 85100, Potenza, Italy*

*** Universiteit Gent, Faculty of Bioscience Engineering, Department of Green Chemistry and Technology, Gr.Karel.de Goedelaan 5, 8500 Kortrijk*

*Corresponding author:* [*marco.cuccarese00@gmail.com*](mailto:marco.cuccarese@unibas.it)

**Table S1**

Correlation parameter of regression, kinetics constant and saturation concentration (theorical and experimental) obtained by the Adams-Bohart fitting of experimental data obtained for atrazine at the different conditions tested.

| Initial concentration  (µg L^-1^) | Bed depth  (cm) | | | Flow rate  (mL min^-1^) | | GTPEG% | | Leaching test | | K  (L min^-1^ µg^-1^) | | N_0_  (µg L^-1^) | N_0exp_  (µg L^-1^) | | R^2^ |
| --- | --- | --- | --- | --- | --- | --- | --- | --- | --- | --- | --- | --- | --- | --- | --- |
| Atrazine | |  |  | |  | |  | |  | |  | | |  |  |
| 500 | 5 | | | 1 | | 5 | | No | | 4⸱10^-6^ | | 15000 | 3040 | | 0.42 |
| 500 | 5 | | | 0.2 | | 5 | | No | | 5⸱10^-7^ | | 20800 | 4400 | | 0.60 |
| 500 | 5 | | | 2.7 | | 5 | | No | | / | | / | 2600 | | 0.02 |
| 500 | 10 | | | 1 | | 5 | | No | | 4⸱10^-6^ | | 8900 | 1600 | | 0.42 |
| 500 | 20 | | | 1 | | 5 | | No | | 2.8⸱10^-6^ | | 5900 | 1000 | | 0.63 |
| 500 | 10 | | | 1 | | 2.5 | | No | | / | | / | 300 | | 0.01 |
| 500 | 10 | | | 1 | | 5 | | Yes | | 3.6⸱10^-6^ | | 9600 | 1800 | | 0.80 |
| 250 | 10 | | | 1 | | 5 | | No | | 4.8⸱10^-6^ | | 9300 | 900 | | 0.46 |
| 125 | 10 | | | 1 | | 5 | | No | | / | | / | 200 | | 0 |

**Table S2**

Correlation parameter of regression, kinetics constant and saturation concentration (theorical and experimental) obtained by the Adams-Bohart fitting of experimental data obtained for carbamazepine at the different conditions tested.

| Initial concentration  (µg L^-1^) | | Bed depth  (cm) | | Flow rate  (mL min^-1^) | | | GTPEG% | Leaching test | | | K  (L min^-1^ µg^-1^) | N_0_  (µg L^-1^) | | N_0exp_  (µg L^-1^) | |  | R^2^ |
| --- | --- | --- | --- | --- | --- | --- | --- | --- | --- | --- | --- | --- | --- | --- | --- | --- | --- |
| Carbamazepine |  | |  | |  |  | | |  |  | | |  | |  |  |  |
| 500 | | 5 | | 1 | | | 5 | No | | | 1.5⸱10^-5^ | 15600 | | 4420 | |  | 0.96 |
| 500 | | 5 | | 0.2 | | | 5 | No | | | 1.4⸱10^-6^ | 20200 | | 7000 | |  | 0.68 |
| 500 | | 5 | | 2.7 | | | 5 | No | | | 1.3⸱10^-5^ | 14200 | | 4000 | |  | 0.62 |
| 500 | | 10 | | 1 | | | 5 | No | | | 1.5⸱10^-5^ | 9200 | | 4200 | |  | 0.96 |
| 500 | | 20 | | 1 | | | 5 | No | | | 1.1⸱10^-5^ | 5500 | | 2100 | |  | 0.60 |
| 500 | | 10 | | 1 | | | 2.5 | No | | | 3.4⸱10^-6^ | 10000 | | 2100 | |  | 0.84 |
| 500 | | 10 | | 1 | | | 5 | Yes | | | 2.4⸱10^-5^ | 8600 | | 4700 | |  | 0.90 |
| 250 | | 10 | | 1 | | | 5 | No | | | 9.4⸱10^-6^ | 9900 | | 1800 | |  | 0.65 |
| 125 | | 10 | | 1 | | | 5 | No | | | / | / | | 500 | |  | 0 |

**Table S3**

Correlation parameter of regression, kinetics constant and adsorption capacity (theorical and experimental) obtained by the Thomas fitting of experimental data for bisphenol A at the different conditions tested.

| Initial concentration  (µg L^-1^) | | Bed depth  (cm) | | Flow rate  (mL min^-1^) | | GTPEG% | | Leaching test | K (L min^-1^ µg^-1^) | | q (µg g^-1^) | | q_exp_ (µg g^-1^) | | R^2^ |
| --- | --- | --- | --- | --- | --- | --- | --- | --- | --- | --- | --- | --- | --- | --- | --- |
| Bisphenol A |  | |  | |  | |  | | |  | |  | |  |  |
| 500 | | 5 | | 1 | | 5 | | No | 3.1⸱10^-5^ | | 103 | | 133 | | 0.89 |
| 500 | | 5 | | 0.2 | | 5 | | No | 6.8⸱10^-6^ | | 76 | | 208 | | 0.47 |
| 500 | | 5 | | 2.7 | | 5 | | No | 1.2⸱10^-4^ | | 40 | | 92 | | 0.54 |
| 500 | | 10 | | 1 | | 5 | | No | 5.6⸱10^-5^ | | 62 | | 96 | | 0.80 |
| 500 | | 20 | | 1 | | 5 | | No | 5.8⸱10^-5^ | | 18 | | 44 | | 0.81 |
| 500 | | 10 | | 1 | | 2.5 | | No | 4.1⸱10^-5^ | | 257 | | 92 | | 0.77 |
| 500 | | 10 | | 1 | | 5 | | Yes | 5.9⸱10^-5^ | | 40 | | 79 | | 0.82 |
| 250 | | 10 | | 1 | | 5 | | No | 1.1⸱10^-4^ | | 27 | | 89 | | 0.90 |
| 125 | | 10 | | 1 | | 5 | | No | / | | / | | 55 | | 0.02 |

**Table S4**

Correlation parameter of regression, kinetics constant and adsorption capacity (theorical and experimental) obtained by the Thomas fitting of experimental data for 17-α ethinylestradiol at the different conditions tested.

| Initial concentration  (µg L^-1^) | Bed depth  (cm) | | | Flow rate  (mL min^-1^) | | GTPEG% | | Leaching test | | K (L min^-1^ µg^-1^) | | q (µg g^-1^) | q_exp_ (µg g^-1^) | | R^2^ |
| --- | --- | --- | --- | --- | --- | --- | --- | --- | --- | --- | --- | --- | --- | --- | --- |
| 17-α ethinylestradiol | |  |  | |  | |  | |  | |  | | |  |  |
| 500 | 5 | | | 1 | | 5 | | No | | 2.3⸱10^-5^ | | 206 | 128 | | 0.51 |
| 500 | 5 | | | 0.2 | | 5 | | No | | / | | / | 292 | | 0.02 |
| 500 | 5 | | | 2.7 | | 5 | | No | | 1.2⸱10^-6^ | | 185 | 111 | | 0.63 |
| 500 | 10 | | | 1 | | 5 | | No | | 5.5⸱10^-5^ | | 62 | 152 | | 0.69 |
| 500 | 20 | | | 1 | | 5 | | No | | 6.6⸱10^-5^ | | 60 | 78 | | 0.75 |
| 500 | 10 | | | 1 | | 2.5 | | No | | 6.1⸱10^-5^ | | 32 | 133 | | 0.87 |
| 500 | 10 | | | 1 | | 5 | | Yes | | 9.1⸱10^-5^ | | 120 | 139 | | 0.87 |
| 250 | 10 | | | 1 | | 5 | | No | | 1.3⸱10^-4^ | | 55 | 139 | | 0.88 |
| 125 | 10 | | | 1 | | 5 | | No | | 9.3⸱10^-5^ | | 44 | 56 | | 0.54 |

**Table S5**

Comparison of the typical parameters of Adams-Bohart and Thomas model obtained for carbamazepine.

| Initial concentration  (µg L^-1^) | | Bed depth  (cm) | | Flow rate  (mL min^-1^) | | GTPEG% | | Leaching test | | K_AB_ (L min^-1^ µg^-1^) | K_Th_ (L min^-1^ µg^-1^) | | q_AB_ (µg g^-1^) | | q_Th_(µg g^-1^) |
| --- | --- | --- | --- | --- | --- | --- | --- | --- | --- | --- | --- | --- | --- | --- | --- |
| Carbamazepine |  | |  | |  | |  | |  | | |  | |  |  |
| 500 | | 5 | | 1 | | 5 | | No | | 1.5⸱10^-5^ | 9.5⸱10^-5^ | | 650 | | 163 |
| 500 | | 5 | | 0.2 | | 5 | | No | | 1.4⸱10^-6^ | 1.5⸱10^-5^ | | 841 | | 236 |
| 500 | | 5 | | 2.7 | | 5 | | No | | 1.3⸱10^-5^ | 6.6⸱10^-5^ | | 592 | | 127 |
| 500 | | 10 | | 1 | | 5 | | No | | 1.5⸱10^-5^ | 9.9⸱10^-5^ | | 383 | | 136 |
| 500 | | 20 | | 1 | | 5 | | No | | 1.1⸱10^-5^ | 7.8⸱10^-5^ | | 229 | | 62 |
| 500 | | 10 | | 1 | | 2.5 | | No | | 3.4⸱10^-6^ | 5.9⸱10^-4^ | | 832 | | 48 |
| 500 | | 10 | | 1 | | 5 | | Yes | | 2.4⸱10^-5^ | 8.7⸱10^-5^ | | 358 | | 160 |
| 250 | | 10 | | 1 | | 5 | | No | | 9.4⸱10^-6^ | 1.1⸱10^-4^ | | 412 | | 51 |
| 125 | | 10 | | 1 | | 5 | | No | | / | 1.3⸱10^-4^ | | / | | 44 |

**Table S6**

Literature overview on removal of carbamazepine, 17-α ethinylestradiol, bisphenol A and atrazine by adsorption on fixed bed of different adsorbent material. References which consider a mix of pollutants in the same solution are evidenced by *. It is important to remember that the influent concentration largely affect the adsorption capacity. By bearing in mind that, comparisons can be done.

| Reference | Adsorbent material | | Initial conditions | | Adsorption capacity, removal | | K, N_0_ | | K_Th,_ adsorption capacity (Thomas model) | |
| --- | --- | --- | --- | --- | --- | --- | --- | --- | --- | --- |
| Carbamazepine | |  | |  | |  | |  | |  |
| Sotelo et al., 2013  Ortinez-Martinez et al., 2018* | Activated carbon  Mesoporous silica  Cu-Amino grafted mesoporous silica | | C_inf_ : 2.5 mg L^-1^  Bed depth: 8 cm=0.8 g  Flow rate: 2 mL min^-1^  C_inf_ : 10 µg L^-1^  Bed depth: 10 cm  Flow rate: 1.5 mL min^-1^  C_inf_ : 10 µg L^-1^  Bed depth: 10 cm  Flow rate: 1.5 mL min^-1^ | | 227 mg g^-1^  45%  0.72 µg g^-1^  0 µg g^-1^ | | Not available  Not available  Not available | | K_Th_: 2.8∙10^-8^ m^3^ µg^-1^ min^-1^  q: 277⸱10^3^  µg g^-1^  Not available  Not available | |
| This work | GTPEG | | C_inf_ : 500 µg L^-1^  Bed depth: 10 cm  Flow rate: 1 mL min^-1^ | | 175 µg g^-1^  42% | | K: 1.5⸱10^-5^ m^3^ µg^-1^ min^-1^  N_0_: 92.3 mg L^1^ | | K_Th_: 9.9⸱10^-5^ m^3^ µg^-1^ min^-1^  q: 136 µg g^-1^ | |
| Bisphenol A | |  | |  | |  | |  | |  |
| Katsigiannis et al., 2015* | Activated carbon | | C_inf_ : 2 µg L^-1^  Bed depth: 4 cm=173 g  Linear velocity: 3 m h^-1^ | | 6⸱10^-2^ µg g^-1^  50% | | K: 9.8⸱10^-8^ m^3^ µg^-1^ min^-1^  N_0_: 22.7⸱10^3^  mg L^-1^ | | Not available | |
| This work | GTPEG | | C_inf_ : 500 µg L^-1^  Bed depth: 10 cm  Flow rate: 1 mL min^-1^ | | 96 µg g^-1^  23% | | K: 5.8⸱10^-6^ m^3^ µg^-1^ min^-1^  N_0_: 92.3 mg L^1^ | | K_Th_: 5.6⸱10^-5^ m^3^ µg^-1^ min^-1^  q: 62 µg g^-1^ | |
| 17-α ethinylestradiol | |  | |  | |  | |  | |  |
| Han et al., 2012 | Aliphatic polyamides | | C_inf_ : 300 μg L^-1^  Bed depth: 2.9 cm=1 g  Contact time: 1 min | | 4470 µg g^-1^ | | Not available | | Not available | |
| De Rudder et al., 2004 | Activated carbon  Manganese oxide | | C_inf_ : 14 μg L^-1^  Flow rate: 20 mL min^-1^  C_inf_ : 14 μg L^-1^  Flow rate: 20 mL min^-1^ | | 55 µg g^-1^  2 µg g^-1^ | | Not available  Not available | | Not available  Not available | |
| This work | GTPEG | | C_inf_ : 500 µg L^-1^  Bed depth: 10 cm  Flow rate: 1 mL min^-1^ | | 152 µg g^-1^  37% | | K: 1.3⸱10^-5^ m^3^ µg^-1^ min^-1^  N_0_: 87.4 mg L^1^ | | K_Th_: 5.5⸱10^-5^ m^3^ µg^-1^ min^-1^  q: 62 µg g^-1^ | |
| Atrazine | |  | |  | |  | |  | |  |
| Jones et al., 1998 | Activated carbon | | C_inf_ : 20 μg L^-1^  Contact time: 1 min | | 104 µg g^-1^ | | Not available | | Not available | |
| This work | GTPEG | | C_inf_ : 500 µg L^-1^  Bed depth: 10 cm  Flow rate: 1 mL min^-1^ | | 65 µg g^-1^  16% | | K: 4⸱10^-6^ m^3^ µg^-1^ min^-1^  N_0_: 89.3 mg L^1^ | | K_Th_: 2.3⸱10^-5^ m^3^ µg^-1^ min^-1^  q: 171 µg g^-1^ | |
|  |  | |  | |  | |  | |  | |
